# Method of Operating in Excision of the Cervix Uteri

**Published:** 1847-02

**Authors:** 

**Affiliations:** Edinburgh


*Method of Operating in Excision of the Cervix Uteri.* By Dr. Simpson, of Edinburgh.—In performing the excision of the cervix uteri, in the three instances I have detailed, and in the other cases in which I have operated, I have proceeded on the following plan:—I have fixed one or two vulsella into the outer or vaginal side of the diseased cervix, as high as it was possible to insert them, and, by the purchase which they afforded, have gradually and cautiously dragged this part down in the lines respectively of the axis of the pelvic brim, cavity, and outlet, till it appeared so far beyond the vulva as to allow me to cut through the base of the protruding cervix. In one or two cases I used a knife in making the incisions. But in consequence of the powerful retraction under which the cervix uteri is placed during the operation, it is difficult, or indeed impossible, to make the incision in this way so equable and perfect as to remove with certainty all the diseased part. After a partial cut or two the uterus is strongly retracted at the points of incision, and the remainder of the operation requires to be finished with the line of incision thus rendered irregular and confused. A pair of large, curved, blunt-pointed scissors, such as were used in this operation by Osiander and Dupuytren, is in this respect preferable. We are enabled by them to surround and embrace the whole cervix at once; and having cautiously and carefully adjusted their edges to the very points which we wish to divide, and thus calculated, at this preliminary step, the exact limits of the incision, we may then immediately complete the amputation of the part, by one or two strong and rapid strokes of the instrument. The blades must be placed around the cervix, *above* the line of the teeth of the vulsellum; and then our object is (as it were) to cut out the vulsellum along with the whole inferior and diseased part of the cervix, in which it is fixed. The operation is much facilitated by the labia being strongly pressed aside by broad copper spatulæ.

I have always placed my patients upon the face, the body being situated across the bed, and the lower extremities hanging over it, as in the operation for hæmorrhoids. We are thus enabled to make our incision through the cervix uteri from behind forwards, instead of from before backwards,—a matter, in my opinion, of no small moment. For if we cut in this latter direction, viz., from *before backwards*, we would sometimes run a greater danger of opening into the peritonæum, which stretches downwards so much more behind than in front of the cervix uteri, and offers a very thin wall of partition between the cavity of the vagina and the cavity of the abdomen. Latterly, I have found the first portion of the operation, namely, the seizure and traction of the cervix uteri, much facilitated by using a very large and strong vulsellum, made with the common loose joint of the obstetric forceps, instead of the usual fixed pivot or scissors joint. With the common scissors-jointed vulsellum, whilst we are intent on fixing the teeth of one blade in a proper situation, the teeth of the other blade are always apt to become entangled in the tumor or walls of the vagina itself, and thus impede and embarrass the operator. But with the modification of the vulsellum that I have alluded to this difficulty is avoided, for the individual blades are introduced, adjusted, and fixed, separately and successively; and then, afterwards, they are easily united together for further use. Besides in this way, we far more readily effect what are, I believe, the two principal secrets in the operation, viz., 1st.: We fix both blades of the instrument, and more especially that corresponding to the diseased lip, as high upon the cervix, and as near its line of reflection upon the roof of the vagina as possible; and 2ndly, by making our line of incision immediately above the hold of the vulsellum (as if our object were to cut out that instrument and the part which it embraces), we secure this important point, that the incision which we make is more likely, than if we followed any other plan, to pass through a stratum of healthy tissue, as we thus inevitably remove the whole vaginal portion of the cervix uteri, and the diseased structure of which it is the seat. In thus attempting to insert the vulsellum as high as possible in the cervix, we will succeed far better by guiding it directly to the point required by the finger and the sense of touch, than by attempting to direct it by the speculum and the sense of sight. In fact, if the cervix is, as generally happens, at all much increased in size, it is, of necessity, utterly impossible to see, with any speculum, the part in which the teeth of the vulsellum should be fixed,—that part lying much higher than the sphere of vision.

Several forms of danger have been found to attend upon excision of the cervix uteri. A fearful variety of nervous depression is alleged by some authors to supervene occasionally upon the operation. I have seen no instance of it. Severe hæmorrhage sometimes occurs, but it is much rarer than we might *a priori* expect. In only one case have I met with it in any considerable amount, and in that instance it was readily and effectually restrained by the plug. Out of nineteen private patients operated on by Lisfranc, Pauly avers that four died within twenty-four hours. Subsequently there is further danger to the patient from inflammation kindling up in some of the uterine structures, or in the peritonæum itself. Out of the eight cases in which I have operated, seven recovered perfectly. The remaining eighth patient recovered so far as to leave her bed-room, but was then, from an unfortunate domestic quarrel, subjected to great mental excitement, after which she relapsed, and died under symptoms resembling those of phlebitis. In his Operative Medicine, Velpeau, after stating that he had himself only operated in two cases, in one of which death occurred in three days, and in the other six weeks after the excision of the cervix, proceeds to remark: "A patient operated upon by M. Blandin died of uterine phlebitis; one of those that Lisfranc lost was carried off by peritonitis; others have sunk under a nervous state, the gravity of which it is not easy to explain. Up to the present time," he continues, "scarcely any one has been seen to die directly of hæmorhage. Rust and Graefe of Berlin, Roux and Dupuytren, who have all seen their patients perish from the immediate results of the operation, do not ascribe the fatal result to this complication (hæmorrhage). Excision of the neck of the uterus, although easy and by no means severe, is nevertheless sometimes extremely dangerous and speedily fatal. However, Osiander has practised it twenty-eight times, Dupuytren fifteen to twenty times, and Lisfranc forty to fifty times, without it having caused death more than once out of every six or seven operated upon."

*Cases adapted for the operation.*—Since excision of the cervix uteri is an operation attended with so many chances of danger, as Velpeau exposes in the passage which I have quoted, it evidently follows that it should only be adopted in cases, and under circumstances, in which milder and safer means of cure are insufficient. The forms of disease in which, upon this principle, it seems justifiable to avail ourselves of the aid of this operation (supposing no contra-indication to be present), are, in my opinion, principally:

1st. Great morbid hypertrophy, by elongation, of the vaginal portion of the cervix uteri. I have operated succesfully in two such cases.

2nd. Corroding ulcer, when limited to the lips of the cervix, and pathologically identical with the form of lupus or malignant ulcer so well known on the face; and

3rd. Circumscribed and local forms of carcinomatous disease or excrescence, of the lips and lower segment of the cervix uteri.

Some continental surgeons, and more especially Lisfranc, advocate the propriety and necessity of excision, in various other cases besides those I have just enumerated. I have myself twice excised the part when affected by chronic induration and thickening, without carcinomatous degeneration; but I would now, most assuredly, by no means resort to it again under the same condition, as I believe that morbid state of the cervix to be quite removable by milder measures. In the case that I have alluded to as having terminated fatally, the structure of the excised cervix presented a great degree of condensation and induration, with two small cystic tumours enclosed in the morbid tissue. The symptoms attendant on this lesion had been of very long standing, and had previously broken down the health of the patient.

Certainly, however, the set of cases in which, of all others, the operation is likely to prove an occasional and important addition to our previous means of treatment, is that in which there exists local carcinoma of the cervix uteri.—*Dublin Quart. Jour. Med. Sci.*

